# The Effect of Nickel on the Microstructure, Mechanical Properties and Corrosion Properties of Niobium–Vanadium Microalloyed Powder Metallurgy Steels

**DOI:** 10.3390/ma13184021

**Published:** 2020-09-10

**Authors:** Mohamed Ahmed Mohamed Ahssi, Mehmet Akif Erden, Mustafa Acarer, Harun Çuğ

**Affiliations:** 1 Department of Mechanical Engineering, Karabuk University, Karabuk 78050, Turkey; ahssimohamed@yahoo.com (M.A.M.A.); hcug@karabuk.edu.tr (H.Ç.); 2 Department of Biomedical Engineering, Karabuk University, Karabuk 78050, Turkey; 3 Department of Metallurgical and Materials Engineering, Selcuk University, Konya 42130, Turkey; macarer@selcuk.edu.tr

**Keywords:** microalloyed steel, powder metallurgy, nickel addition, microstructure, mechanical properties, corrosion

## Abstract

In this study, the effects of adding Ni in different ratios to Fe-matrix material containing C-Nb-V produced by powder metallurgy on microstructure, tensile strength, hardness and corrosion behaviors were investigated. Fe-C and Fe-C-Nb-V powders containing 5%, 10%, 13%, 15%, 20%, 30% and 40% nickel were pressed at 700 MPa and then sintered in an Ar atmosphere at 1400 °C. Microstructures of the samples were characterized with optical microscope, scanning electron microscope (SEM) and XRD. Corrosion behaviors were investigated by obtaining Tafel curves in an aqueous solution containing 3.5% NaCl. Mechanical properties were determined by hardness and tensile testing. While Fe-C alloy and Fe-C-Nb-V microalloyed steel without Ni typically have a ferrite-pearlite microstructure, the austenite phase has been observed in the microstructures of the alloys with 10% nickel and further. Yield and tensile strength increased with nickel content and reached the highest strength values with 13% Ni content. The addition of more nickel led to decrease the strength. Analysis of Tafel curves showed that corrosion resistance of alloys increased with increasing nickel concentration.

## 1. Introduction

Microalloyed steels are known as steels containing elements such as titanium, vanadium and aluminum at a maximum of 0.20% by wt. These steels are a group of materials with superior properties such as excellent durability, weldability, corrosion resistance and toughness achieved by applying different strength enhancer mechanisms and appropriate thermomechanical procedures [1,2].

In the literature, powder metallurgy (PM) is defined as a method of producing small, functional, incompatible, difficult-to-manufacture parts such as composite structures, with high strength and minimum tolerance, in a more advantageous and economical way compared to other production methods. Therefore, many industrial components such as automotive, defense, health and energy sectors are produced by PM [3,4,5].

Nickel is an austenite stabilizer and widens the austenite region and contracts the ferrite region in steel. Nickel improves the resistance against the corrosion and oxidation at elevated temperatures. Nickel improves the toughness and strength by refining the grain size. It also prevents scale forming on the material surface. When used with chromium, it improves the hardness, ductility, fatigue resistance and critical cooling rate. Elemental nickel has a lesser diffusion coefficient compared to many elements and diffuses into iron more slowly [6,7].

In metallurgy, materials science and mechanical engineering, there is a lot of research about the relationship between the production of PM steel, its microstructure, its mechanical properties and its corrosion behaviors. Some of them have indicated the usefulness of powder metallurgy. Erden [5] produced Nb-V microalloyed PM steel. He investigated the effects of the sintering temperature on tensile and hardness behaviors of the PM steel. He figured out that the sintering temperature is the best at 1350 °C. The author also determined that Nb-V bearing to steel caused an increase in the tensile and hardness strengths of the PM steels. Turkmen et al. [8] examined the effects of TiN additions on tensile behaviors of PM steels. Results indicated that the addition of TiN in the percentage of 0.1, 0.2 or 0.5 increases tensile strength properties of the powder metal parts.

Demirtaş and Erden [9] produced Cr-Ni steel with the PM method. In this study, the effects of Cr–Ni addition on the tensile strength properties of powder metallurgy plain carbon steel were investigated. For this purpose, Cr-Ni (wt.% 0.5–3) was added to the matrix containing 0.5 C with powder metallurgy method. The tensile test, microhardness measurement and microstructure analysis were fulfilled to investigate the effect of Cr-Ni additions on the tensile behavior. As a result, it was determined that the added alloying elements increased the mechanical properties, but the mechanical properties decreased with the amounts of alloying elements increasing after 1 wt.%

Though the advantages of Ni addition on the tensile, hardness and corrosion behaviors of steels have been documented, few studies have examined the effects of Ni on the composition of the Fe-C-NbV microalloyed PM steel. As PM is an important production technique for steels, Ni-containing PM steels could be needed in the future to maintain the compatibility between the PM advantages such as net shape production or without machining, and mechanical and corrosion properties of these steels. The main objective of this study was to explore the effects of adding up to 40 wt.% Ni to Fe-C-NbV alloy on the mechanical and corrosion properties and microstructure.

## 2. Materials and Methods

**Figure 1:**
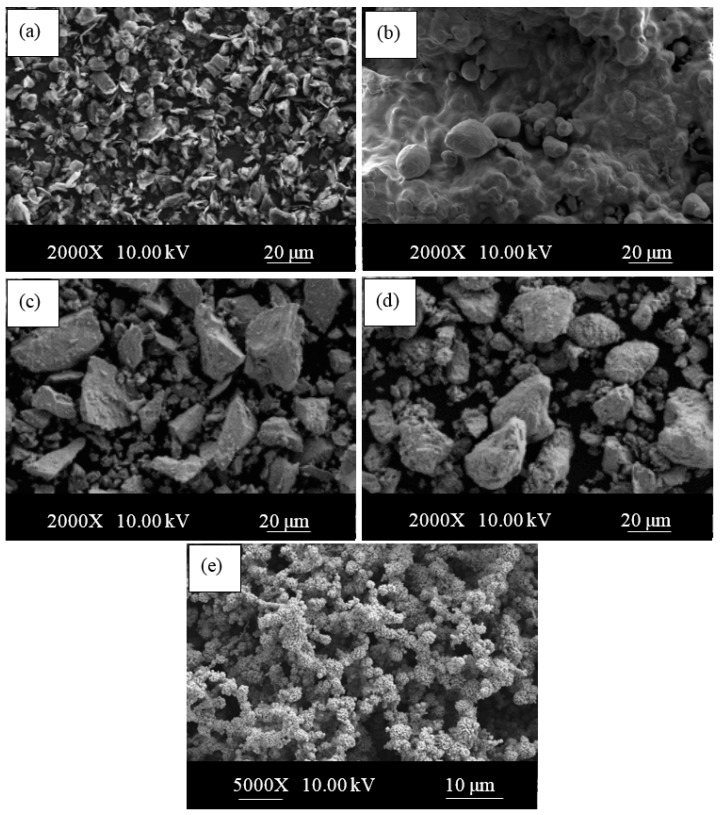
SEM micrographs of powders; (**a**) graphite, (**b**) iron, (**c**) niobium, (**d**) vanadium and (**e**) nickel.

In this study, the microstructure, tensile, hardness and corrosion behaviors of Fe-C and Fe-C-Nb-V-xNi alloys containing different Ni percentages produced by powder metallurgy were investigated. The sizes and purities of graphite, iron, niobium vanadium and nickel powders used in the study were <20, ≤180, <45, <44 and 5 μm, and 96.5%, 99.9%, 99.8%, 99.5% and 99.7%, respectively. (Figure 1).

Before the mixing process, to weigh the powders, RADWAG AS-60-220 C/2 with a precision scale at 10^−4^ g, trademark was used. In Table 1, the chemical compositions of the powders can be seen. Fe-C-NbV-xNi powders were mixed for an hour by using a TURBULA T2F device (Willy A. Bachofen AG, Muttenz, Switzerland) that works on three-dimensional motion principle. The powders were pressed to form the tensile test specimen’s shape according to the ASTM E8/E8M [10], using a hydraulic press (Hidroliksan, Konya, Turkey) with 100 ton capacity by applying 750 MPa pressing pressure. After pressing, sintering was carried out in Argon atmosphere at 1400 °C for 2 h. Radwag density kit (Bruker Alpha, Bursa, Turkey) using the Archimedes principle based on ASTM B 328-96 [11] to determine the densities of the specimens. Densities and chemical compositions of the alloys are listed in Table 1.

Optical microscopy studies were carried out with Nikon ECLIPSE L150 type microscope (Melville, NY, USA). Grain sizes of alloys were determined via the mean linear intercept method [12,13]. Zeiss microscope and Rigaku Ultima IV diffractometers were used for SEM and XRD, respectively, for microstructural and fracture surface examinations. XRD was used for the qualitative analysis of the structure of the alloys changing with adding Ni.

A SHIMADZU hardness test device (MCT-W, Shimadzu, Tokyo, Japan) was used to determine the microhardnesses of these parts under HV_0.5_ load. The alloys were exposed to tensile tests at 1 mm/min crosshead speed using a SHIMADZU tensile test device having 50 kN capacity (Shimadzu, Tokyo, Japan).

Electrochemical potentiodynamic tests were applied to determine the corrosion performances of the alloys in 3.5% NaCl solution. A “Parstat 4000 (Rotalab, Ankara, Turkey)” was used as a potentiostat with an electrochemical cell in which a saturated calomel electrode (SCE) was used as a reference electrode. After 3 h of immersion of the samples, potentials were applied between −0.5 and 0.5 V versus open circuit (O.C.) potentials with a scan rate of 2.5 mV /s. Here, anodic and cathodic regions were scanned separately (the O.C. potential was chosen as the initial scanning point, so O.C. potentials are equivalent to corrosion potentials) in order to maintain more reliable surfaces of the working electrodes through the measurements of corrosion currents and stabilize the effects of applied potentials. In our setup, we tried our best to place capillary lugging as close to working electrode as possible. This setup should reduce any extra voltage drop. Before the measurements the surfaces of the samples were prepared with a series of different meshes of abrasive papers (400, 600, 800, 1000, 1200 and 2000 meshes, coarse to fine). Then all of them were cleaned by ethyl alcohol and distilled water.

## 3. Results

**Figure 2:**
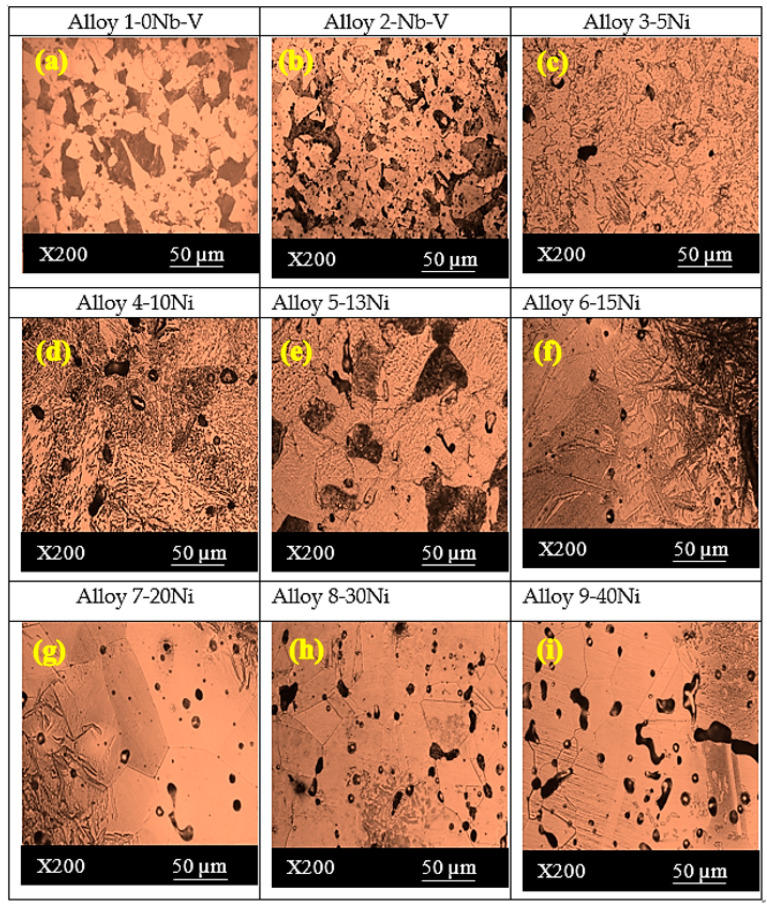
The optical micrographs of the alloys (**a**) without Nb–V, (**b**) without Ni, (**c**) 5.0 wt.% Ni, (**d**) 10 wt.% Ni, (**e**) 13 wt.% Ni, (**f**) 15 wt.% Ni, (**g**) 20 wt.% Ni, (**h**) 30 wt.% Ni, (**i**) 40 wt.% Ni.

Alloy 1 and 2 having perlite and ferrite phases are shown in Figure 2. Microalloying elements can be added as single, double or triple combinations to achieve the desired tensile and hardness strength in the microalloyed steels. In the present study, the addition of 0.15% Nb-V resulted in a decrease in the grain size compared with Fe-C alloy without Nb-V (Figure 2).

**Figure 3:**
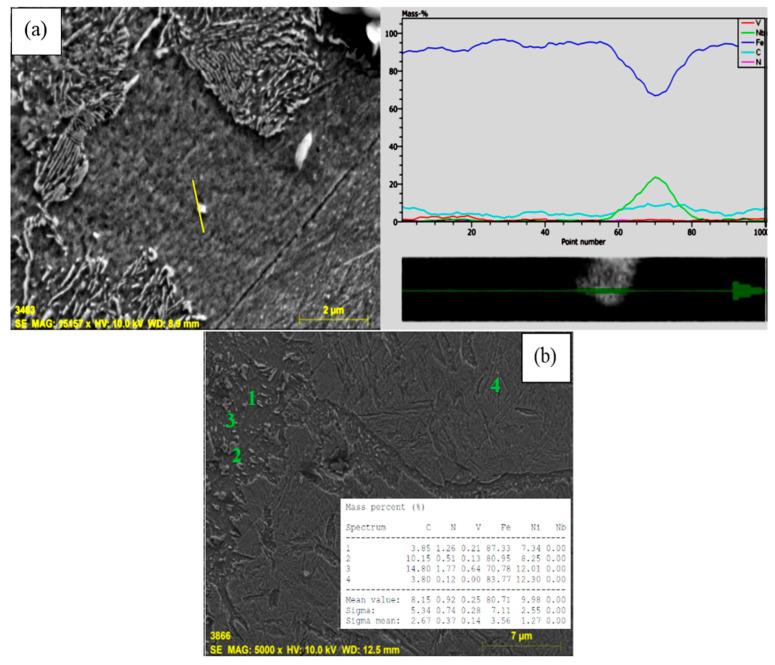
(**a**) SEM line EDS image of alloy 2 and (**b**) precipitates formed at grain boundaries.

This may be the result of the creation of small precipitates such as niobium carbonitride (NbCN) and vanadium carbonitride (VCN) during the sintering at 1400 °C or during the post-sintering cooling process. The EDS analysis indicated that Nb and V formed into grains and grain boundaries in the solution as precipitates (Figure 3a,b). The EDS line analysis also catches out that the type and number of elements in Nb–V microalloyed PM steels without Ni (alloy 2) varied along the line intersecting with the matrix and precipitates (see Figure 3). The precipitates observed in alloys via SEM and EDS analyses are known to have important effects upon recrystallization and austenite grain growth [14,15,16]. The microalloying elements such as Nb and V precipitate as vanadium carbide (VC), vanadium nitrite (VN), vanadium carbonitride (VCN), niobium carbide (NbC), niobium nitrite (NbN) and niobium carbonitride (NbCN) during the hot rolling or sintering followed by cooling for PM process, contributing to the desired tensile and hardness strength of microalloyed steels by grain refinement, solid solution hardening and precipitation hardening mechanisms [14,15,16,17,18,19,20]. When the average grain sizes of alloy 1 and alloy 2 are calculated, while the average grain size of alloy 1 was 36.8 µm, it could be seen that the average grain size of alloy 2 decreased to 30.5 µm due to forming precipitates such as VC(N) and NbC (N) during sintering. The precipitates prevented the austenite grains growth [7,21,22,23,24].

**Figure 4:**
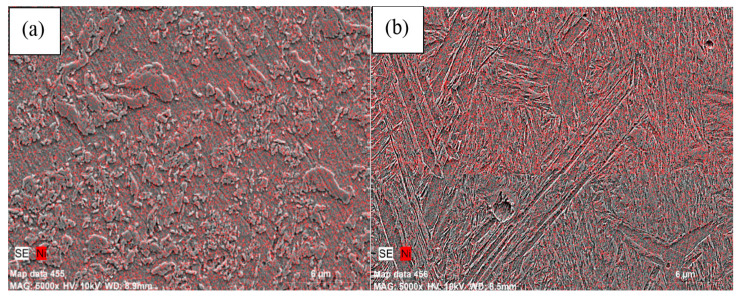
Elemental mapping for (**a**) alloy 3 and (**b**) alloy 4 sintered at 1400 °C.

In the microstructures of alloys containing 5% and 10% Ni, bainitic structures in which cementite lamellars are shorter than those of pearlite and the austenite grains were observed in the microstructure (see Figure 2). Austenite grains became more apparent in alloys containing 13 wt.% Ni. Besides, inhomogeneous microstructures appeared with increasing Ni contents. As can be seen from the mapping analyses for alloy 3 and alloy 4 given in Figure 4, Ni segregated locally with the increase of Ni amount from 5% to 10% due to lower diffusion coefficient of Ni (see Figure 4). Elemental nickel has a low diffusion coefficient compared to many elements, such as carbon, copper and molybdenum, and thus it diffuses into the iron very slowly and this led to insufficient distribution during the sintering process, as stated by Tracey and Upadyaya in their studies [6,7].

While alloy 5-13Ni alloy has a pearlite structure with austenite grains, a needle-shaped martensitic structure has locally segregated next to austenite grains in alloy 6-15Ni alloy. In the alloy 7-20Ni alloy, a slightly martensitic structure appears next to austenite grains, while the structure consists entirely of austenite grains in alloy 8-30Ni and alloy 9-40Ni alloys.

The post-sintering densities of the samples are around 93% due to porosities. Porosities may occur in alloys produced by powder metallurgy [18]. The densities slightly increased after the sintering process. The findings of similar studies support that the density increases with the sintering process [5,14,22,23,24]. The contact points expand, and the formation of the neck occurs, and the pores are reduced between the powder particles during sintering. Grain boundaries are created in the regions of the neck. The grain boundaries expand, and the material gets a more homogeneous structure. In this study, it was observed that the porosity increased with the increase of nickel in the microstructure images of the alloys. Alloy 8-30Ni and alloy 9-40Ni have more porosities than other alloys with lower Ni content. From here, it is possible to obtain the knowledge that the presence of different alloy elements and their size and shape differences make it difficult to press and sinter to low alloys.

**Figure 5:**
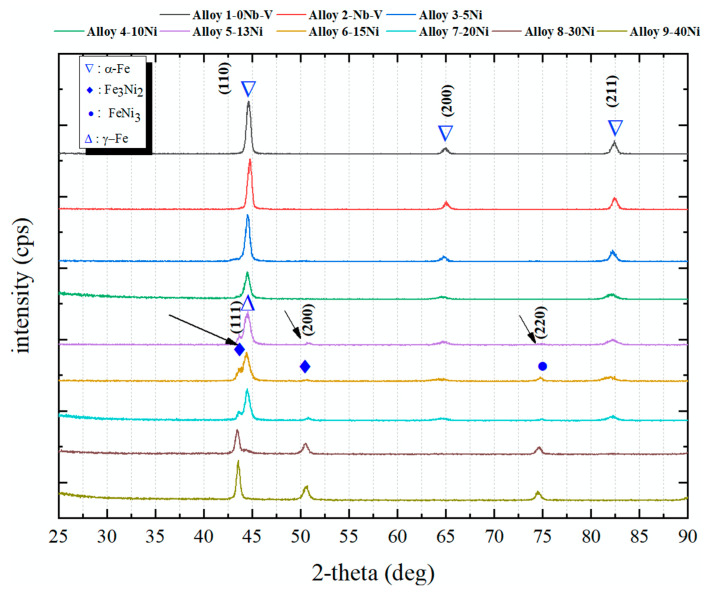
XRD patterns of the alloys.

To characterize the microstructure further, XRD measurements were fulfilled on the alloys with and without Ni. Figure 5 shows the XRD patterns of the alloys. While alloy 1-0Ni, alloy 2-NbV and alloy 3-5Ni alloys containing 5% Ni respectively give similar peaks, the peaks at 2-theta 65° and 82.5° are damped with increasing Ni content. The peak seen in 44.6° shifted to the left. In the alloys containing 13% and over Ni, new peaks started to appear at 50.78° and 74.82°. For the alloys containing 20% and above of Ni, new peaks are formed at 43.60° and the peak at 44° of the alloy containing 40% Ni is completely damped. In high Ni contents, the peaks at 43° and 44° to the left indicate that the structure has transformed into FCC and the Fe3Ni compound may have formed [25]. Additionally, the presence of new peaks at b+ were found for the structure as well [25,26].

**Figure 6:**
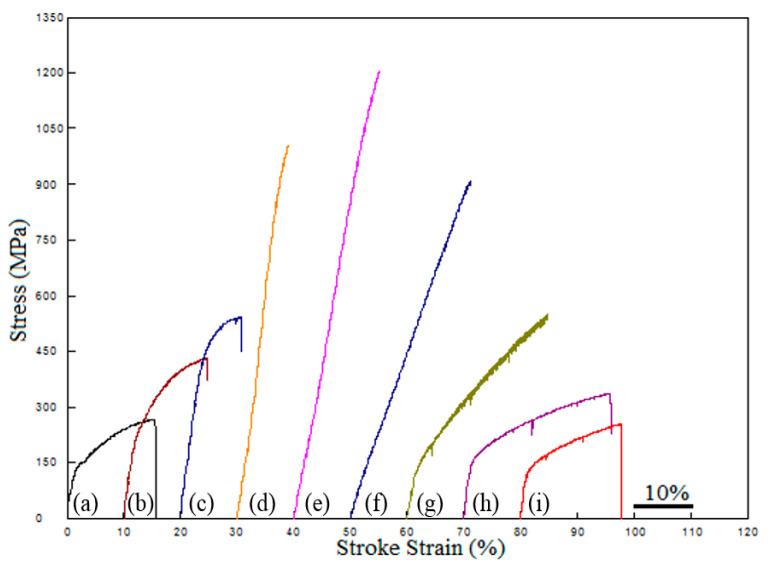
The stress–strain curves for powder metallurgy specimens at different percentages of Ni content (a—alloy 1, b—alloy 2, c—alloy 3, d—alloy 4, e—alloy 5, f—alloy 6, g—alloy 7, h—alloy 8, and i—alloy 9).

Figure 6 and Table 2 list the hardness, yield strength (YS), ultimate tensile strength (UTS) and ductility in terms of the elongation at fracture of the alloys with and without Ni addition. When comparing Figure 6 and Table 2 in terms of mechanical properties, graphite ratios in alloy 1 and 2 are kept constant and the effects of Nb and V are examined. It is clear that the mechanical properties of alloy 2 which is produced by adding Nb and V to alloy 2 are better than those of alloy 1. Nb and V elements form precipitates during the sintering process and later, which improves the YS and UTS through strength improving mechanisms such as precipitation hardening and grain size refinement. Similar studies also support these results. Erden produced Nb-V microalloyed steel by PM method sintering at different temperatures. They sintered the samples at different temperatures (1150, 1250, 1350 and 1400 °C) for 60 min under an argon environment and determined that increasing Nb-V ratio (0.1–0.2%) increases the YS and UTS. They attributed this increase to the formation of precipitates such as NbC(N) and VC(N) during and after the sintering. He also determined that Nb-V microalloyed steel has better mechanical properties when sintered at 1350 °C [5,14,15,16]. Furthermore, the yield point elongation of nonalloyed PM steels was longer than that of microalloyed PM steel. This demonstrated that VC (N) and NbC (N) precipitates were formed in the microalloyed steel and the amounts of free C and N atoms in the solid solution caused a reduction in the yield point elongation [27]. Yield point elongation depends on the density of free atoms such as carbon and nitrogen that prevent the dislocations. Precipitation of carbides and nitrides reduces the atom density (C and N) around the dislocations leading to a decrease in yield point elongation. With the addition of 13 wt.% Ni, major increases in hardness and strength with respect to those of the other Ni contents and Ni-free could be obtained. The addition of Ni over 13% caused a decrease in strength, while the elongation values reached 26%. This shows the microstructural variations caused by the Ni addition and in agreement with the microstructural observations. In the literature [28,29], in studies on determining the effect of Ni addition on the mechanical properties of steels, it is seen that the high Ni content in post-quenching processes decreases the strength as it causes residual austenite. Additionally, in powder metallurgy studies [30,31] it has been reported to decrease strength, as excess alloy content causes porosity. However, in studies without heat treatment, the addition of Ni has increased the strength somewhat.

An increasing amount of added Ni is thought to cause formations of bainitic and martensitic structures. Getting et al. investigated the effects of Ni addition on the mechanical properties of molybdenum PM steels. They reported that an increasing amount of Ni addition improves the mechanical properties of the produced PM steels such as hardness and tensile strength. They also reported that the microstructure of molybdenum steel without Ni is formed by ferrite and pearlite, and addition of nickel to 0–2% by weight produces harder phases in the microstructure. If nickel is added 2–5% by weight, bainitic and martensitic structure exist in the structure [6]. Element nickel has a low diffusion coefficient compared to many elements such as carbon, copper and molybdenum, and thus it diffuses into the iron very slowly [6,7].

**Figure 7:**
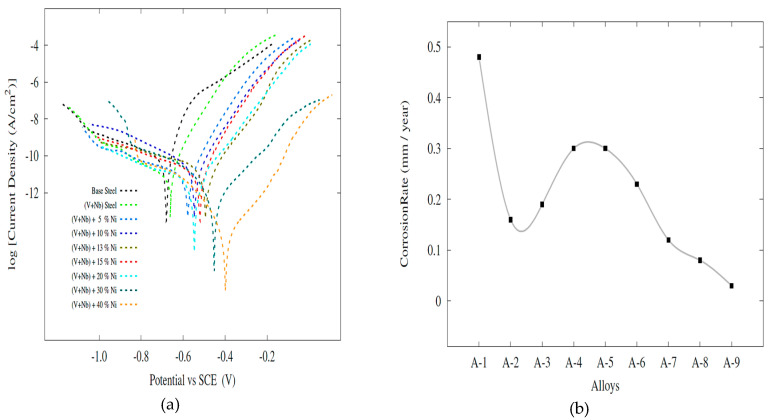
Tafel curves (**a**) and corrosion rate curves (**b**) the alloys.

The Tafel curves (Figure 7) and the Ecorr and Icorr values (Table 3) obtained from the potentiodynamic corrosion tests and corrosion rates were calculated using the relevant formulae given in ASTM-G102 [32]. Mass transport effects are not apparent from the figures. Additionally, a change in the mechanism of reaction for most of the scanning regions does not seem to appear (except for anodic region of base steel, yet that region is mostly out of fitting region). Therefore, we believe that Tafel description of overall corrosion dynamics should be possible. It seems that addition of Nb and V into pure steel leads to a significant increase of corrosion resistance [33,34,35,36,37]. There are many studies in the literature that say microalloyed steels have good corrosion resistance compared to unalloyed steels. Uygur et al. [33] examined the corrosion properties of unalloyed steel and microalloyed steel with different ratios of Nb–V microalloying elements by powder metallurgy. The results obtained have shown that the corrosion resistance increases almost three times with the addition of 0.075 Nb and V by weight of unalloyed steel. The results obtained are similar to those in this study. The grain size has decreased as a result of the addition of Nb–V to unalloyed steels. It is informed us that the fine pearlite ferrite structure is more resistant to corrosion than the coarse pearlite ferrite structure. The particle size reduction is beneficial for the creation of dense passive films. [35,36]. Grain size refinement’s effects on corrosion resistance were investigated in the literature and similar conclusions were reached. Ura-Bińczyk et al. [36] informed us that grain refinement causes a more intense and uniform passive film on the N80-1 steel surface. Thus, it has been observed that N80-1 steel is more corrosion resistant than K55 steel.

Nickel addition into this microalloyed steel, however, leads to a relatively complex behavior. Comparing Ni addition to Nb-V microalloyed steel, nickel in amounts up to 15 percent seems to offer no advantage from the standpoint of corrosion resistance [36]. Figure 7 shows lower corrosion resistance with increasing Ni content for alloys with concentrations lower than 10 wt.% Ni. However, this behavior dramatically reversed for alloys with Ni concentrations higher than 13 wt.% Ni. Further addition of Ni into NbV microalloyed steel converts these alloys to high corrosion resistant metallic systems, evident also from shifting of corrosion potentials (not systematically though) (Table 3). Surprisingly, however, we observed increased corrosion resistance after further addition of Ni element (above 10 wt.% Ni).

When Figure 7 is examined, the best corrosion resistance is seen in steel containing 40% nickel by weight. Actually, it was expected that an increase would be in the corrosion resistance, with increasing Ni content. However, due to martensitic structures in the alloys 3, 4i, 5 and 6, corrosion resistance decreased. In the literature, there are studies supporting these results [36,37,38,39]. Pettibone [38] demonstrated an advantage in the corrosion resistance of 36.53 Ni wt.% steel over mild steel in four sea water environments for exposure periods varying from 5 to 15 years. It has also been reported that the corrosion rate of a 26 percent Ni alloy of iron and nickel was about one third that of wrought iron in sea water and in the atmosphere [39]. Alharthi et al. has investigated the effect of the amount of nickel on the corrosion resistance of Fe-36% Ni and Fe-45% Ni alloys in 1M hydrochloric acid pickling solution. The EIS results showed that the polarization and surface resistances for the alloy with high Ni content were much higher. The results showed that the Fe-45% Ni alloy has much better corrosion resistance than the Fe-36% Ni alloy [18]. Pavapootanont et al. [17] investigated the corrosion properties of aerated steels containing 15% Ni, 23% Ni, 31% Ni and 40% Ni in 3.5% NaCl solutions with different pH values (pH 2, 7, 10) at 25 °C. Although both pitting potentials and primary passive potentials of these three steels increased with the increase in the amount of nickel, the passive current density reduced. Nickel has developed general corrosion and pitting corrosion resistances of the tested steels in all three solutions. However, results of XRD analysis (Figure 5) give much more insight to the observed corrosion performance vs. Ni addition.

**Figure 8:**
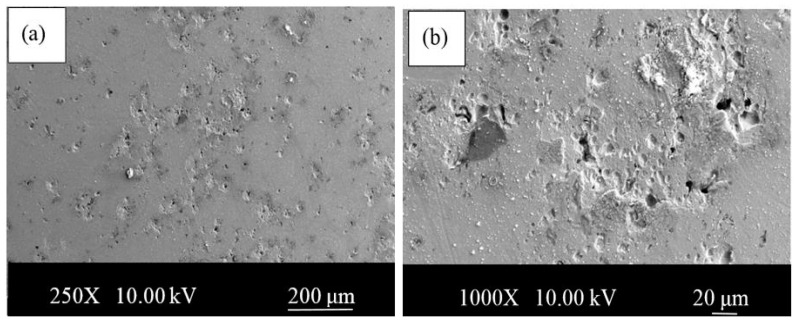
SEM images of the alloy 2-NbV-0Ni after corrosion (**a**) low and (**b**) high magnification.

**Figure 9:**
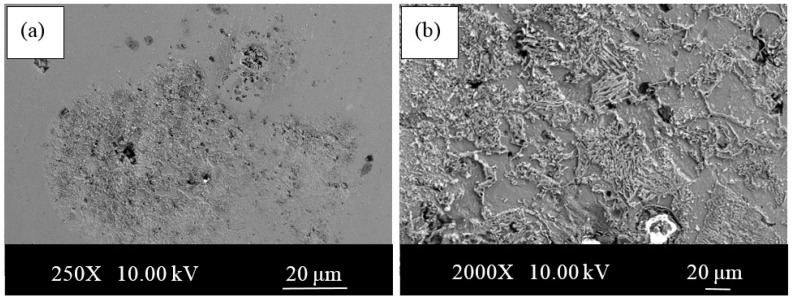
SEM images of the alloy 3-NbV-5Ni after corrosion (**a**) low and (**b**) high magnification.

**Figure 10:**
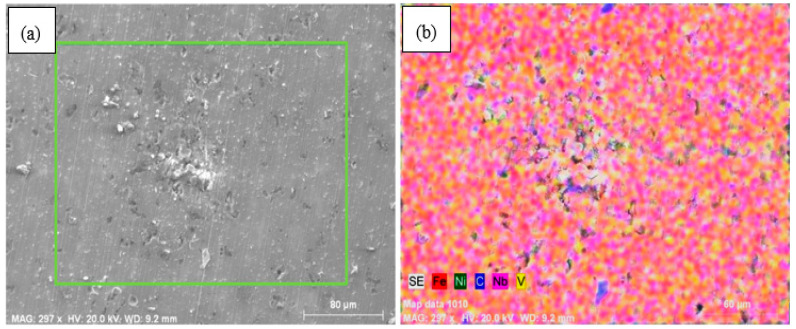
(**a**) SEM image and (**b**) mapping analysis of the alloy 2-NbV-0Ni after corrosion.

**Figure 11:**
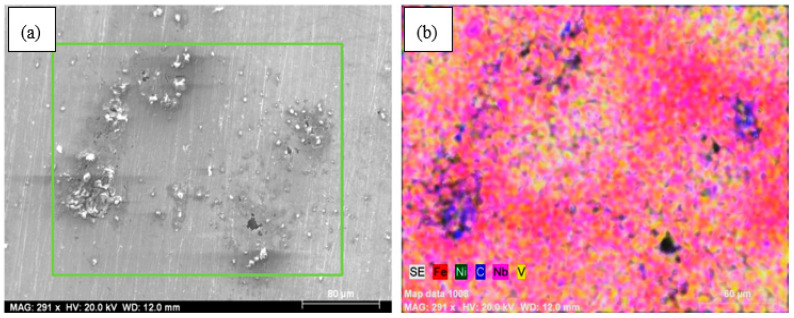
(**a**) SEM image and (**b**) mapping analysis of the alloy 9-NbV-40Ni after corrosion.

It seems that under 10 wt.% addition of Ni there is no Fe3Ni phase. Above 10 wt.% Ni addition there is a clear appearance of Fe3Ni phases (at theta1, theta2, etc.), which sharply correlates with corresponding corrosion performance. SEM images of the samples with Ni and without Ni after corrosion test are given in Figure 8 and Figure 9. Whereas the samples without Ni have corrosion scales on the surface, it was observed that pittings localized on the alloy 3-NbV-5Ni after corrosion. Figure 10 and Figure 11 shows SEM image and mapping analysis of alloy 2-NbV-0Ni and alloy 9-NbV-40Ni respectively.

**Figure 12:**
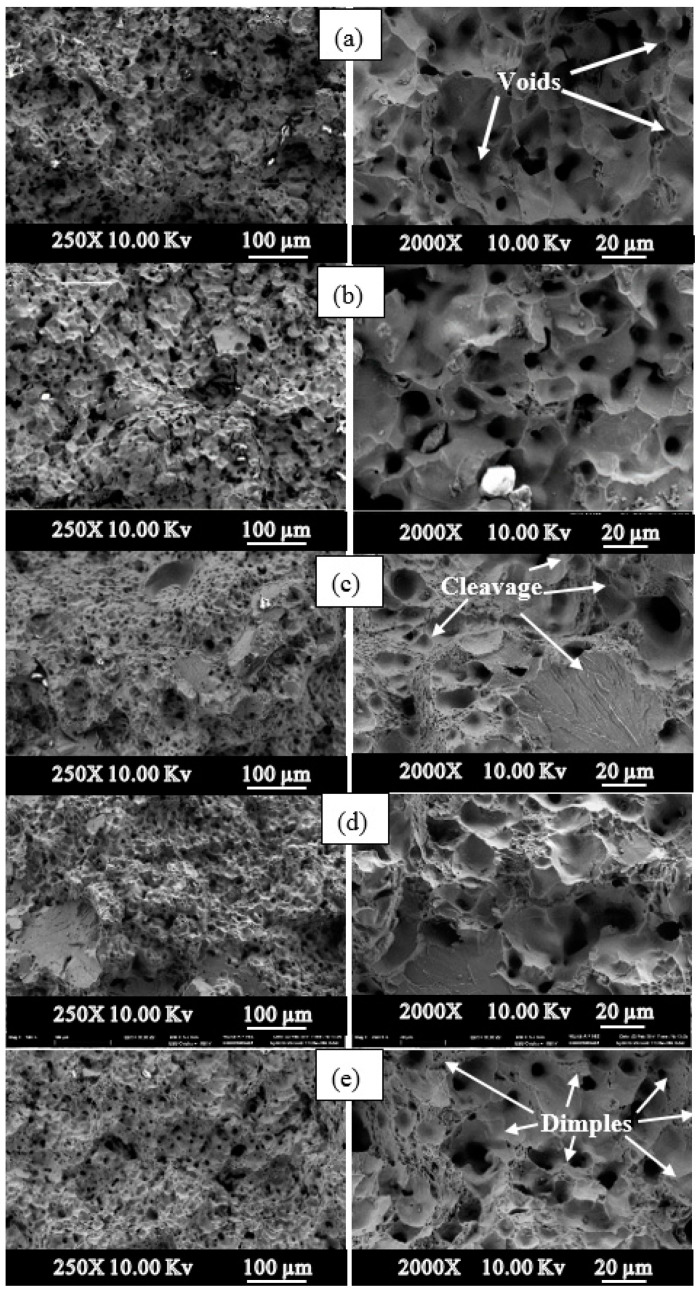
Fracture surfaces of PM steels (250×–2000×). (**a**) Alloy 1, (**b**) Alloy 2, (**c**) Alloy 4, (**d**) Alloy 5, (**e**) Alloy 8).

As seen in Figure 12, when the SEM pictures of steels containing different amounts of Ni are examined, the fracture surfaces have partially ductile (honeycomb structure) and partially brittle (cleavage plane) structures. Voids are visible across the entire fracture surface. This situation shows that fractures spread by the coalescence of microvoids. Ceavage planes, however, were most prominent in alloy 2 and least prominent in alloy 1. In addition, large gaps were observed in the TM steel samples produced. The presence of these voids indicates that precipitates such as VC (N) and NbC (N) are detached from the surface during tensile testing. In his study, Shanmugasundaram and Chandramouli [40] saw that such large gaps were formed on the broken surfaces of TM steel containing Cr, Ni and Mo, and this was due to the separation of carbides from the surface during the tensile test. As a result of the tensile test, with the increase of Ni amount, increases in YS and UTS, and a decrease in % elongation were observed. The cracked surface pictures are compatible with the values obtained as a result of the tensile test. When the SEM images of samples with different nickel compositions were examined (Figure 12), they showed that the broken surfaces were completely partially brittle and partially ductile. It was clearly seen that pores were present on all of the broken surfaces. This situation shows that the breakage is realized by the joining and progression of microvoids. However, the separation planes, which are indicative of brittle fracture, appear to be the highest in the steel sample containing 10% Ni by weight; medium in the nonalloy and microalloy steel samples without Ni; and the least in the steel sample containing 30% Ni by weight.

## 4. Conclusions

In this study, Fe-C-NbV-xNi alloys fabricated by powder metallurgy with and without Ni were characterized through microstructural observations, mechanical tests and corrosion tests. The following conclusions have been drawn.

1. The microstructures of the alloys with nonalloyed steel and Nb–V microalloyed steel without Ni have ferrite and pearlite. However, increasing Ni content caused the transformation to austenite and the formation of Fe_3_Ni_2_ FeNi FeNi_3_ intermetallic compounds.
2. Ni addition up to 13% increased the yield and tensile strength with hardness. More addition of that decreased the strength but increased the elongation.
3. Ni content increased the corrosion resistance almost systematically, although it was also low in the alloys with 5%, 10%, 13% and 15% Ni due to possible microstructure inhomogeneity and martensitic structure.

## Figures and Tables

**Table 1:** Chemical components of powder metallurgy specimens.

Sample	C (wt.%)	Nb (wt.%)	V (wt.%)	Ni (wt.%)	Fe (wt.%)	Densities (g/cm^3^)
Alloy 1-0Ni	0.55	-	-	-	Rest	7.2837
Alloy 2-NbV-0Ni	0.55	0.075	0.075	-	Rest	7.2787
Alloy 3-NbV-5Ni	0.55	0.075	0.075	5	Rest	7.4102
Alloy 4-NbV-10Ni	0.55	0.075	0.075	10	Rest	7.2347
Alloy 5-NbV-13Ni	0.55	0.075	0.075	13	Rest	7.5947
Alloy 6-NbV-16Ni	0.55	0.075	0.075	16	Rest	7.6724
Alloy 7-NbV-20Ni	0.55	0.075	0.075	20	Rest	7.5484
Alloy 8-NbV-30Ni	0.55	0.075	0.075	30	Rest	7.2903
Alloy 9-NbV-40Ni	0.55	0.075	0.075	40	Rest	7.2231

**Table 2:** Mechanical properties of the compositions.

Alloy	YS (MPa)	UTS (MPa)	Elongation (%)	Hardness (Hv 0.5)
Alloy 1-0Ni	140	270	16	75
Alloy 2-NbV-0Ni	220	435	15	125
Alloy 3-NbV-5Ni	390	545	11	160
Alloy 4-NbV-10Ni	555	1010	10	243
Alloy 5-NbV-13Ni	630	1205	16	244
Alloy 6-NbV-15Ni	480	910	21	191
Alloy 7-NbV-20Ni	135	552	25	133
Alloy 8-NbV-30Ni	155	340	26	93
Alloy 9-NbV-40Ni	118	259	18	78

**Table 3:** Ecorr and Icorr values of the alloys.

	Specimen	Ecorr (V)	Icorr (μA/cm^2^)	CR (mm/year)
A-1	Pure Steel	−0.68	37.9	0.48
A-2	(V + Nb) Steel	−0.66	12.5	0.16
A-3	(V + Nb) Steel + 5% Ni	−0.58	14.9	0.19
A-4	(V + Nb) Steel + 10% Ni	−0.54	23.8	0.30
A-5	(V + Nb) Steel + 13% Ni	−0.49	23.9	0.30
A-6	(V + Nb) Steel + 15% Ni	−0.52	18.3	0.23
A-7	(V + Nb) Steel + 20% Ni	−0.54	9.3	0.12
A-8	(V + Nb) Steel + 30% Ni	−0.45	6.12	0.08
A-9	(V + Nb) Steel + 40% Ni	−0.40	2.3	0.03

## References

[1] Gladman T. (1997). The Physical Metallurgy of Microalloyed Steels.

[2] Gündüz S., Karabulut H., Erden M.A., Türkmen M. (2013). Microstructural Effects on Fatigue Behaviour of a Forged Medium Carbon Microalloyed Steel. Mater. Test..

[3] Erden M.A. (2015). An Investigation on the Relation Sheep Between Microstructure and Mechanical Properties of Microalloyed Steels Produced by Powder Metallurgy. Ph.D. Thesis.

[4] Jing W., Yisan W., Yichao D. (2008). Reaction synthesis of Fe–(Ti,V)C composites. J. Mater. Process. Technol..

[5] Erden M.A. (2017). The Effect of the Sintering Temperature and Addition of Niobium and Vanadium on the Microstructure and Mechanical Properties of Microalloyed PM Steels. Metals.

[6] Tracey V. (1992). Nickel sintered steels—Developments, status and prospects. Met. Powder Rep..

[7] Upadhyaya G.S. (2000). Sintered Metallic and Ceramic Materials-Sintered Low-Alloy Ferrous Materials.

[8] Türkmen M., Karabulut H., Erden M.A., Gündüz S. (2017). Effect of TIN Addition on The Microstructure and Mechanical Properties of PM Steels. E-J. New World Sci. Acad..

[9] Demirtaş H., Erden M.A. (2019). The Effect of Cr and Ni Addition on Mechanical Properties of Plain Carbon Steel. Düzce Uni. J. Sci. Technol..

[10] ASTM E8/E8M (2013). Standard Test Methods for Tension Testing of Metallic Materials.

[11] ASTM B328-96 (2004). Standard Test Method for Density, Oil Content, and Interconnected Porosity of Sintered Metal Structural Parts and Oil-Impregnated Bearings.

[12] Fischmeister H.F. (1972). Applications of quantitative microscopy in materials engineering. J. Microsc..

[13] Gladman T., Woodhead J. (1960). The accuracy of point counting in metallographic investigation. J. Iron Steel Inst..

[14] Erden M.A., Gündüz S., Türkmen M., Karabulut H. (2014). Microstructural characterization and mechanical properties of microalloyed powder metallurgy steels. Mater. Sci. Eng. A.

[15] Türkmen M., Erden M., Karabulut H., Gündüz S. (2019). The Effects of Heat Treatment on the Microstructure and Mechanical Properties of Nb-V Microalloyed Powder Metallurgy Steels. Acta Phys. Pol. A.

[16] Gündüz S., Erden M.A., Karabulut H., Türkmen M. (2016). Effect of the addition of niobium and aluminium on the microstructures and mechanical properties of micro-alloyed PM steels. Mater. Teh..

[17] Pavapootanont G., Wongpanya P., Viyanit E., Lothongkum G. (2018). Corrosion Behavior of Ni Steels in Aerated 3.5-wt.% NaCl Solution at 25 °C by Potentiodynamic Method. Eng. J..

[18] Alharthi N., Sherif E.-S.M., Abdo H.S., El Abedin S.Z. (2017). Effect of Nickel Content on the Corrosion Resistance of Iron-Nickel Alloys in Concentrated Hydrochloric Acid Pickling Solutions. Adv. Mater. Sci. Eng..

[19] Sage A.M. An overview of the use of mikroalloys in HSLA steels with particular reference to vanadyum and titanium, processing, properties and applications. Proceedings of the Second International Conference on HSLA Steels.

[20] Jung J.-G., Park J.-S., Kim J., Lee Y.-K. (2011). Carbide precipitation kinetics in austenite of a Nb–Ti–V microalloyed steel. Mater. Sci. Eng. A.

[21] Sepúlveda R., Arenas F. (2001). TiC–VC–Co: A study on its sintering and microstructure. Int. J. Refract. Met. Hard Mater..

[22] Erden M.A., Gündüz S., Karabulut H., Türkmen M. (2016). Effect of vanadium addition on the microstructure and mechanical properties of low carbon micro-alloyed powder metallurgy steels. Mater. Test..

[23] Huo X.-D., Mao X., Lü S.-X. (2013). Effect of Annealing Temperature on Recrystalization Behavior of Cold Rolled Ti-Microaloyed Steel. J. Iron Steel Res. Int..

[24] Erden M.A. (2020). The Effect of Sintering Time on Tensile Strength of NB-V Microalloyed Powder Metallurgy Steels. E-J. New World Sci. Acad..

[25] Noskov A.I., Gilmutdinov A.K., Yanbaev R.M. (2017). Effect of coaxial laser cladding parameters on bead formation. Int. J. Miner. Met. Mater..

[26] Wang L., Zhou J., Yu Y., Guo C., Chen J. (2012). Effect of powders refinement on the tribological behavior of Ni-based composite coatings by laser cladding. Appl. Surf. Sci..

[27] Lou S., Northwood D.O. (1995). Effect of temperature on the lower yield strength and static strain ageing in low-carbon steels. J. Mater. Sci..

[28] Norwood C.G. (2018). The Effect of Nickel Content on the Mechanical Properties and Microstructure of a High Toughness Secondary Hardening Steel. Ph.D. Thesis.

[29] Aparova A.I., Lyapunov A.I., Eremin V. (1987). Effect of nickel and manganese on the structure and properties of steel R3M3F2. Met. Sci. Heat Treat..

[30] Özdemirler D., Gündüz S., Erden M.A. (2017). Influence of NbC Addition on the Sintering Behaviour of Medium Carbon PM Steels. Metals.

[31] Behera D., Tripathi P., Chaubey A. (2018). Effect of Nickel on Mechanical Properties of Alloy Steel Produced by Powder Metallurgy. Mater. Today Proc..

[32] ASTM G102-89 (2015). Standard Practice for Calculation of Corrosion Rates and Related Information from Electrochemical Measurements.

[33] Uygur İ., Gerengi H., Erden M.A., Yıldız M. (2017). The Effect of Niobium and Vanadium on Corrosıon Of Low Carbon Steel Obtained By Powder Metallurgy In 3.5%Nacl Envıronment. Tech. App. Sci..

[34] Huang L.-Y., Wang K., Wang W., Zhao K., Yuan J., Qiao K., Zhang B., Cai J. (2019). Mechanical and corrosion properties of low-carbon steel prepared by friction stir processing. Int. J. Miner. Met. Mater..

[35] Ura-Bińczyk E., Dobkowska A., Płocińska M., Płociński T., Adamczyk-Cieślak B., Mazurkiewicz B., Solarski W., Banaś J., Mizera J. (2017). The influence of grain refinement on the corrosion rate of carbon steels in fracturing fluids used in shale gas production. Mater. Corros..

[36] Schwerdtfeger W. (1966). Corrosion rates of binary alloys of nickel and iron measured by polarization methods. J. Res. Natl. Bur. Stand. Sect. C Eng. Instrum..

[37] Bai Q., Zou Y., Kong X., Gao Y., Dong S., Zhang W. (2017). The influence of the corrosion product layer generated on the high strength low-alloy steels welded by underwater wet welding with stainless steel electrodes in seawater. J. Ocean Univ. China.

[38] LaQue F.L., Copson H.R. (1963). Corrosion Resistance of Metals and Alloys.

[39] Marsh J.S. (1938). Alloys of Iron and Nickel.

[40] Shanmugasundaram D., Chandramouli R. (2009). Tensile and impact behaviour of sinter-forged Cr, Ni and Mo alloyed powder metallurgy steels. Mater. Des..

